# Transcriptome Analysis Unveils Gln3 Role in Amino Acids Assimilation and Fluconazole Resistance in *Candida glabrata*

**DOI:** 10.4014/jmb.2012.12034

**Published:** 2021-04-21

**Authors:** Francisco J. Pérez-de los Santos, Luis Fernando García-Ortega, Karina Robledo-Márquez, Jesús Guzmán-Moreno, Lina Riego-Ruiz

**Affiliations:** 1 Unidad de Genómica Avanzada, Laboratorio Nacional de Genómica para la Biodiversidad (Langebio), CINVESTAV, Mexico; 2 División de Biología Molecular, Instituto Potosino de Investigación Científica y Tecnológica A. C. (IPICYT), Mexico; 3 Departamento de Ingeniería Genética, Centro de Investigación de Estudios Avanzados del IPN (CINVESTAV), Mexico

**Keywords:** *Candida glabrata*, nitrogen catabolite repression, Gln3, ABC transporters, fluconazole resistance

## Abstract

After *Candida albicans*, *Candida glabrata* is one of the most common fungal species associated with candidemia in nosocomial infections. Rapid acquisition of nutrients from the host is important for the survival of pathogens which possess the metabolic flexibility to assimilate different carbon and nitrogen compounds. In *Saccharomyces cerevisiae*, nitrogen assimilation is controlled through a mechanism known as Nitrogen Catabolite Repression (NCR). NCR is coordinated by the action of four GATA factors; two positive regulators, Gat1 and Gln3, and two negative regulators, Gzf3 and Dal80. A mechanism in *C. glabrata* similar to NCR in *S. cerevisiae* has not been broadly studied. We previously showed that in *C. glabrata*, Gln3, and not Gat1, has a major role in nitrogen assimilation as opposed to what has been observed in *S. cerevisiae* in which both factors regulate NCR-sensitive genes. Here, we expand the knowledge about the role of Gln3 from *C. glabrata* through the transcriptional analysis of BG14 and *gln3*Δ strains. Approximately, 53.5% of the detected genes were differentially expressed (DEG). From these DEG, amino acid metabolism and ABC transporters were two of the most enriched KEGG categories in our analysis (Up-DEG and Down-DEG, respectively). Furthermore, a positive role of Gln3 in AAA assimilation was described, as was its role in the transcriptional regulation of *ARO8*. Finally, an unexpected negative role of Gln3 in the gene regulation of ABC transporters *CDR1* and *CDR2* and its associated transcriptional regulator *PDR1* was found. This observation was confirmed by a decreased susceptibility of the *gln3*Δ strain to fluconazole.

## Introduction

Fungi are a major group of eukaryotic organisms with an estimated number of species ranging from 3.5 to 5.1 million [1]. Among these, only a small fraction has the capacity to cause invasive fungal infections. Some of the fungal species recognized as causing agents of candidemia in nosocomial infections are *Candida albicans*, *Candida parapsilosis* and *Candida glabrata* [2, 3]. Commonly, candidemia is treated with different azole antifungal agents, fluconazole being the primary therapeutic option. However, infections caused by *C. glabrata* have been reported to exhibit varying levels of intrinsic or acquired fluconazole resistance [4-8]. The mechanisms of resistance include an increased drug efflux for cell detoxification [9]. This resistance is mainly mediated through members of the ABC family transporters, Cdr1 and Cdr2/Pdh1, which are activated by the transcriptional regulator Pdr1 [10-13].

Pathogenic fungi have different ways to survive and divide inside the host where growth conditions are not optimal. Rapid acquisition of nutrients from the host are important for pathogens, such as *C. glabrata*. This yeast has the metabolic flexibility to assimilate different carbon and nitrogen compounds, to persist, grow, and survive within the host. Nitrogen assimilation has been well studied in various fungi, like in the yeast *Saccharomyces cerevisiae* and in the filamentous fungi *Aspergillus nidulans* and *Neurospora crassa*. These organisms selectively utilize preferred nitrogen sources (*e.g.* glutamine or ammonium), instead of non-preferred ones (*e.g.* proline or GABA). Preferential utilization of the available nitrogen sources is achieved through a similar mechanism known as Nitrogen Catabolite Repression (NCR, in *S. cerevisiae*) and Nitrogen Metabolite Repression (NMR, in *A. nidulans* and *N. crassa*) [14-16]. In *S. cerevisiae*, NCR is coordinated by the action of four GATA factors; two positive regulators, Gat1 and Gln3, and two negative regulators, Gzf3 and Dal80. These GATA factors recognize the consensus sequence 5’-GATAAG-3’ in the promoter region of their target genes [14, 15]. We have previously shown that in *C. glabrata*, Gln3 has an important role in nitrogen assimilation. The absence of Gln3 affected cell growth when glutamine, ammonium or proline were used as nitrogen sources [17]. Furthermore, we demonstrated that this GATA factor regulates *MEP1*, *GAP1*, and *GLN1* (encoding the ammonium permease, the general amino acid permease and the glutamine synthetase, respectively) expression when assessed in preferred and non-preferred nitrogen sources (ammonium and proline, respectively) [17]. However, the role of Gln3 in NCR regulation of *C. glabrata* is far from being well understood. Here, we decided to explore and compare the transcriptomes of parental (BG14) and *gln3*Δ strains when grown in ammonium as the only nitrogen source. We report the whole-transcriptome profile of a *gln3*Δ mutant of *C. glabrata*, which prompted us to investigate the role of Gln3 in aromatic amino acids assimilation and in fluconazole resistance.

## Materials and Methods

### Strains and Growing Media

Parental (BG14; *ura3*::Tn903 Neo^R^), *gln3*Δ (*ura3*::Tn903 Neo^R^, *gln3*Δ::FRT) and *pdr1*Δ (*ura3*::Tn903 Neo^R^, *pdr1*Δ::*hph*) strains were previously described [17, 18]. YPD rich media was prepared with 10 g/l^-1^ yeast extract (BD; Bioxon, USA), 20 g/l^-1^ peptone (BD; Bioxon) and 2% (w/v) glucose (J. T. Baker). Minimal media (MM) was prepared with 1.7 g/l^-1^ of yeast nitrogen base (YNB) without amino acids and (NH_4_)_2_SO_4_ (BD; Difco), 2% (w/v) glucose (J. T. Baker), and 1 mg/ml^-1^ of the indicated nitrogen source; additionally, 30 μg/ml^-1^ of uracil (Sigma-Aldrich, USA) was added to fulfill yeast auxotrophy. Nitrogen sources and uracil used were filter sterilized and added to the media after autoclaving. For solid media, 2% (w/v) agar (Sigma-Aldrich) was added. Cell cultures were routinely grown at 30°C with constant shaking at 220 rpm.

### RNA-seq Sample Preparation, Library Construction and Sequencing

Six independent yeast cultures (3 for BG14 and 3 for *gln3*Δ strains) were grown overnight in YPD media on a roller drum with constant spinning. Cells were washed twice and resuspended in 1 ml of sterile milliQ water. Flasks containing 50 ml of MM were inoculated at a final optical density at 600 nm (OD_600nm_) of 0.1, and incubated at 30°C with constant shaking (220 rpm) until OD_600nm_ of 0.8 to 1.0 was reached. Cells were pelleted, rapidly transferred to liquid nitrogen and stored at -80°C. RNA extraction, sequencing library construction and sequencing were performed by the Beijing Genomics Institute (BGI, China). Sequencing libraries were sequenced using the Illumina HiSeq 2000 platform and paired-end reads with an average length of 100 bp were obtained. Raw data were deposited in the NCBI Short Read Archive (SRA) under accession no. PRJNA498279.

### Quality Filtering and De Novo Assembly of the Reads

After sequencing, adapters were removed from the reads using cutadapt v1.9.1 [20], using the sequences reported by Illumina for TruSeq RNA v2/LT and paired-end DNA oligonucleotides. Reads were filtered by their quality value using Trimmomatic v0.36 software [21], under the parameters “HEADCROP:12 SLIDINGWINDOW:4:24 MINLEN:50. De novo assembly of the filtered reads was performed using Trinity v2.2.0 [22], with the --jaccard_clip and --SS_lib_type (RF) parameters to minimize fusion transcripts.

### Abundance Estimate and Annotation of the Transcripts

To estimate the expression abundance of the assembled transcripts, reads were re-mapped to the assembled contigs using bowtie2 v2.2.6, keeping only the reads uniquely mapped to one unigene. Then, read counts for each transcript were estimated using RSEM v3 [23, 24]. Only transcripts with expression evidence of at least one correctly aligned read were retained for subsequent analysis. Assembled contigs were identified by the best BLAST hit against *C. glabrata* CBS138 peptides and UniProt non-redundant proteins databases [25-27]. Only hits with an expected value less than 1 × 10^-6^ were considered significant. Although the BG14 strain is derived from BG2 strain and not from CBS138 strain, we used the information of the latter in the annotation process due to the quality of its genome annotation and its phylogenetic closeness (Fig. S1). GO, KEGG and FunCat enrichment analysis were performed using clusterProfiler package of R [28], with a custom database of *C. glabrata* made with Annotation Forge package. Only categories with *q*-value < 0.05 were considered as significant.

### Undetected Gene Estimation, PCA, and Differential Gene Expression Analysis

To estimate the number of undetected genes, a non-parametric estimator h6 was used [29], while the expected number of genes was calculated as the sum of the number of observed and undetected genes. We used the prcomp R function of the stats package to perform Principal Component Analysis (PCA) [30]. The cumulative variation of the two principal components was approximately 99.82%. The exactTest function of the edgeR package was used to determine gene differential expression [31]. The resulting *p*-values were corrected with the *q*-value function using the default parameters to obtain an FDR of 5% [30, 32].

### Growth Curve Experiments

Yeast cultures were incubated overnight in liquid YPD media on a roller drum with constant spinning. Then, cells were washed twice with sterile milliQ water and inoculated to an OD_600nm_ of 0.1 in MM supplemented with any of the following nitrogen sources: ammonium (J. T. Baker), histidine (Sigma-Aldrich), tryptophan (Sigma-Aldrich), tyrosine (Sigma-Aldrich) and phenylalanine (Sigma-Aldrich) at a final concentration of 1 mg/l^-1^. Growth curves were performed in biological triplicates in a Bioscreen C MBR machine at 30°C with constant shaking fixed at maximum. OD_600nm_ measurements were taken every 15 min during 24 h, with the exception of YPD growth curves in which OD_600nm_ measurements were taken every 30 min.

### Fluconazole Susceptibility Assays

BG14, *gln3*Δ and *pdr1*Δ strains were grown in YPD at 30°C until stationary phase. Then, cultures were washed twice with sterile milliQ water and kept in milliQ water during 1 h at room temperature. After this, OD_600nm_ was adjusted to 0.5 in milliQ water and serial dilutions were prepared (from 10^0^ to 10^-4^), and spotted on solid MM with ammonium (1 mg/ml^-1^) buffered with MOPS (0.165 M) to pH 6.5, containing different fluconazole (Pfizer) concentrations (2, 4, 6, 8, 16, 32, 64, 128 or 256 μg/ml^-1^). The plates were photographed after incubation for 48 h (BG14 and *pdr1*Δ strains) and 72 h (*gln3*Δ strain) at 30°C.

### Quantitative PCR (qPCR)

Yeast cultures were grown overnight in YPD media on a roller drum with constant spinning. Then, cells were washed twice and resuspended in 1 ml of sterile milliQ water. Cell cultures were inoculated at OD_600nm_ of 0.1 in flasks containing 50 ml of MM and incubated at 30°C with constant shaking (220 rpm) until OD_600nm_ of 0.8 to 1.0 was reached (mid-log phase). Cells were pelleted and total RNA extracted as previously described [19]. For cDNA synthesis, RNA was first treated with Turbo DNase (AMBION); next, cDNA was synthesized using a Superscript II (Invitrogen, USA) Reverse Transcriptase Kit with oligo(dT)_18_. qPCR was performed using SYBR Green Master Mix (Applied Biosystems) in a Piko Real 96 Real-Time PCR System (Thermo-Scientific, USA). CAGL0K12694g (*ACT1*) gene expression was used as normalization control. All primers used in the present work are listed in Additional File 1.

## Results

### Transcriptome Assembly and Gene Expression Changes

**Fig. 1:**
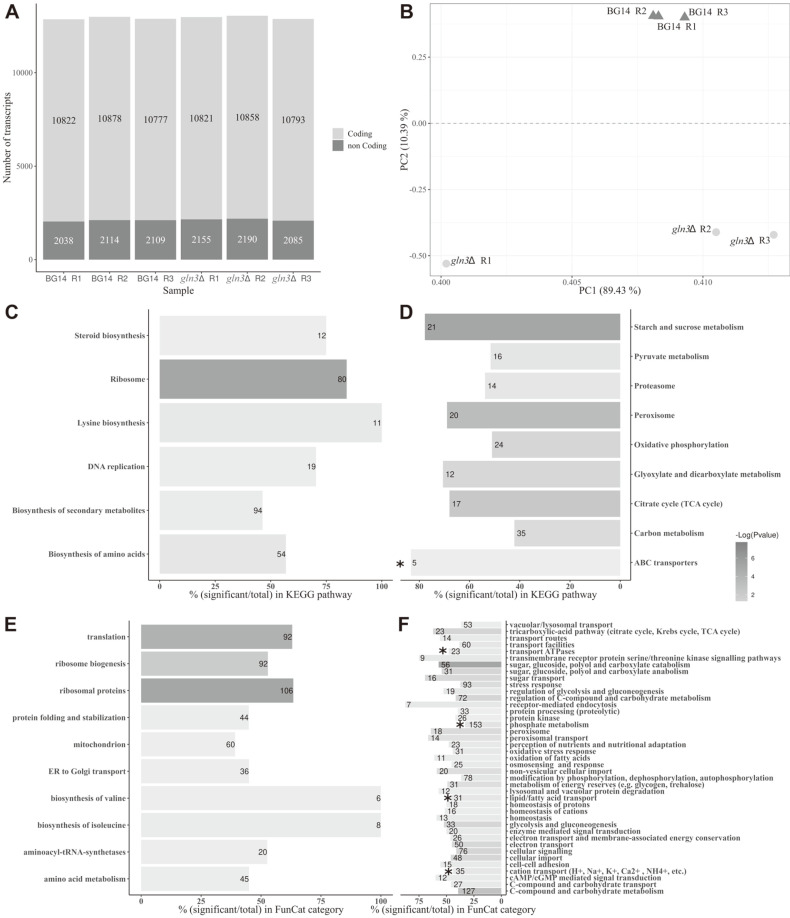
Enrichment analysis results for Differentially Expressed Genes (DEG) according to expression profile. (**A**) Proportions of coding and non-coding transcripts per sample. (**B**) Principal components analysis (PCA) of the six libraries sequenced. (**C**) Enrichment KEGG pathways for genes with higher expression in a BG14 compared to a *gln3*Δ strain (Up-DEG). (**D**) Enrichment KEGG pathways for genes with higher expression in a *gln3*Δ than in a BG14 strain (Down-DEG). (**E**) Enrichment FunCat categories for Up-DEG. (**F**) Enrichment FunCat categories for Down-DEG. The x-axis shows the number of differentially expressed genes (stated by the number on each bar) over the total number of genes in the category (by percentage). Gray scale of the bars represents the significance of the *q* value. Dark gray tones correspond to low values and light gray tones correspond to high values. Bars with KEGG and FunCat categories that include *CDR1* are denoted with an asterisk. No significant categories were found that included *PDR1*.

To investigate how many genes are under Gln3 regulation when *C. glabrata* cells are grown in ammonium as sole nitrogen source, RNA-seq analysis was performed in BG14 and *gln3*Δ strains. In total, 51,757,566 raw reads were generated. From these, 44,837,461 (86.63%) sequences were considered as high-quality reads which were then assembled de novo in 14,501 transcripts. An average of 10,824 coding and 2,115 non-coding transcripts were found (Fig. 1A and Table 1), providing evidence of expression of 4,415 genes (Additional File 2). To test the gene detection rate of our study, the number of undetected genes was calculated. There was an average of 7 undetected genes per sample (0.15%), which indicates the high gene detection rate of our study (99.85% of the expected genes). Transcriptome sequencing data reported in this study are available under PRJNA498279 bioproject accession number, at the NCBI Sequence Read Archive (SRA), and the gene expression data are available in Additional File 3.

From the 4,415 detected genes, 2,362 (53.50%) were Differentially Expressed Genes (DEG) (Additional File 4). Among these, 1,171 genes were upregulated (Up-DEG) in the BG14 strain, suggesting a positive regulation of these genes by Gln3, while 1,191 genes were downregulated (Down-DEG), showing a putative negative regulation by Gln3. Principal component analysis (PCA) for transcript expression was performed. We observed a similar global expression profile for BG14 samples within the same group, whereas *gln3*Δ replicates were not all closely grouped in the first component (Fig. 1B). PCA is highly sensitive to outlying observations and may not capture the variation of the regular observations [33]. However, the estimated common dispersion value in our data was lower (0.0016) to that observed in technical replications on genetically identical model organisms (0.01) [34]. So, we considered this variation as marginal and all transcriptomic data obtained from the *gln3*Δ biological triplicates were used for the analysis (Fig. 1B).

**Fig. 2:**
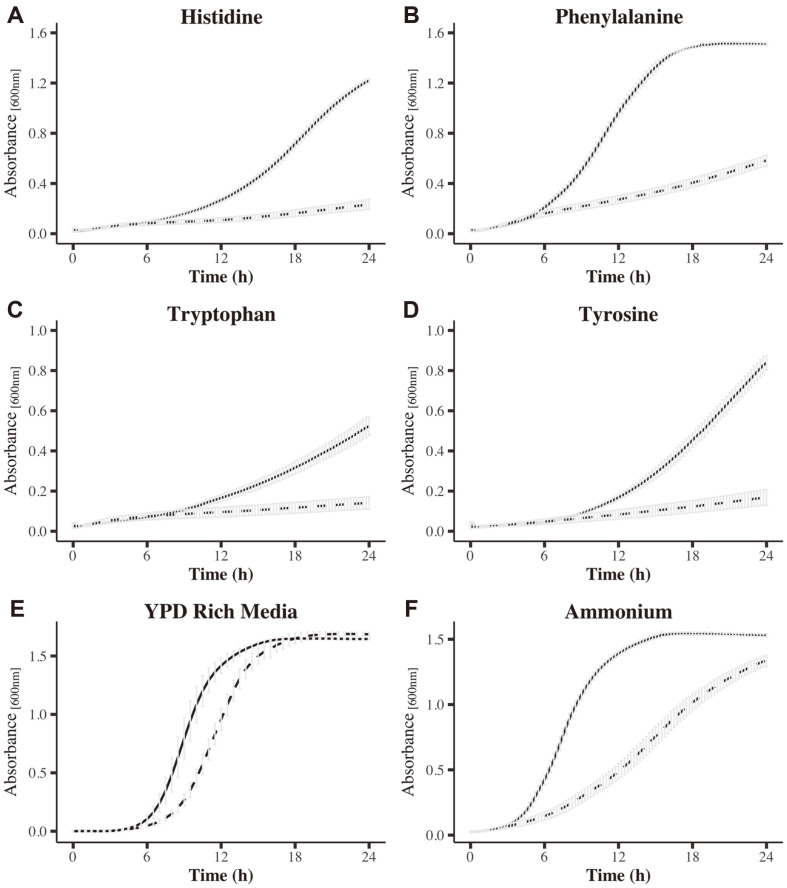
Growth curves of BG14 and *gln3*Δ strains of *C. glabrata* in different nitrogen sources. Yeast cultures of BG14 (solid lines) and *gln3*Δ (dashed lines) were grown in MM media supplemented with the indicated nitrogen source at a final concentration of 1 mg/ml^-1^ (**A-D** and **F**). (**E**) YPD rich media was used as growth control. For each curve, results are depicted as the mean standard deviation from three independent experiments.

**Gln3 is necessary for growth on aromatic amino acids (AAA) and histidine.** Previously, we reported an important role of Gln3 for nitrogen assimilation in a biosynthetic media with ammonium as nitrogen source [17]. Here, our data shows that in addition to our previous report, several other genes corresponding to the KEGG pathway of amino acids biosynthesis (Figs. 1C and 1E; Additional File 5), are also positively regulated by Gln3. Among those genes is *ARO8* (CAGL0G01254g), which encodes for the aromatic amino transferase I. From the two aromatic amino transferases (Aro8 and Aro9) described in *C. glabrata*, Aro8 has been shown to be crucial for proper histidine and aromatic amino acids (AAA; phenylalanine, tryptophan and tyrosine) utilization as nitrogen sources [35, 36]. In addition, *ARO8* expression is higher than *ARO9* in the above-mentioned nitrogen sources [35, 36]. Thus, we decided to investigate if Gln3 is involved in the utilization of histidine and AAA, through the transcriptional regulation of *ARO8*. First, we compared the growth of BG14 and *gln3*Δ strains in MM with histidine or AAA as nitrogen sources. As Fig. 2 shows, absence of Gln3 affected growth on rich medium (YPD) and ammonium, as previously reported [17]. In addition, the *gln3*Δ growth was severely affected in histidine and any of the AAA as nitrogen sources (Figs. 2A−D), generating a phenotype that strongly resembles the one observed in an aro8Δ mutant strain when grown under similar conditions [35]. These results suggest that Gln3 could be acting as a regulator of Aro8. In fact, *ARO8* expression was found to be Gln3 dependent when ammonium was used as a nitrogen source (Fig. S2), but not in cells from cultures grown on histidine or AAA (Fig. S3). These data indicate that Gln3 is necessary for proper growth of *C. glabrata* on both biosynthetic and catabolic conditions (*i.e.* ammonium and amino acids, respectively), and that *ARO8* is the target of its regulation under biosynthetic but not on catabolic conditions.

**Fig. 3:**
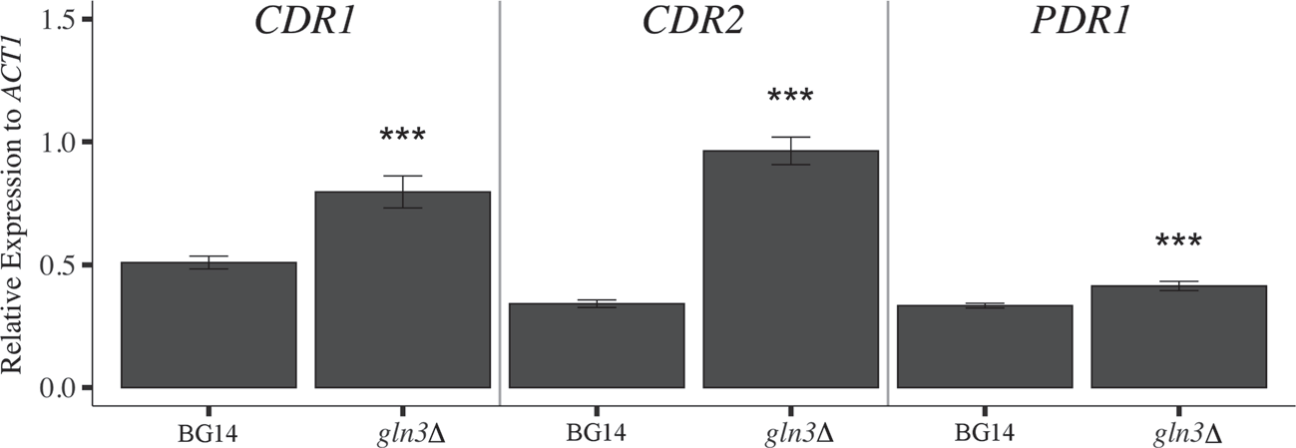
Gln3 is a negative regulator of *CDR1*, *CDR2* and *PDR1* gene expression in *C. glabrata*. Gene expression of *CDR1*, *CDR2* and *PDR1* was determined in 5 μg of total RNA extracted from yeast cultures on MM with ammonium as sole nitrogen source. The reported gene expression is relative to *ACT1* and was measured through qPCR. Relative gene expression represents the mean of three independent experiments and two technical replicates standard deviation. ***Gene expression is significantly different from that calculated in the BG14 strain, alpha < 0.001 in a two-tailed *t*-test.

**The ABC transporters involved in drug resistance were highly expressed in a *gln3*Δ mutant.** The differential gene expression analysis showed the enrichment of a functional KEGG category, ABC transporters (Figs. 1D and 1F; Additional File 5). Some of these transporters are involved in cell detoxification from drugs, specifically azoles. We decided to investigate a possible role of Gln3 in fluconazole resistance, through the transcriptional regulation of the ABC transporter genes *CDR1* and *CDR2*, and the transcriptional regulator *PDR1*. In accordance with our transcriptome analysis, qPCR from yeast cultures grown in MM with ammonium as a nitrogen source, showed that *CDR1*, *CDR2*, and *PDR1* gene expression was negatively dependent on Gln3, suggesting an unrecognized role of Gln3 in azole resistance (Fig. 3). So, we evaluated *CDR1* and *CDR2* expression in the BG14 and *pdr1*Δ strains. We observed that *CDR1* expression, but not *CDR2*, was Pdr1 dependent (Fig. S4). Our results are consistent with previous findings which showed that Cdr1 is the main efflux pump implicated in the detoxification of yeast cells after fluconazole exposure; while the effect of Cdr2 is perceptible only when Pdr1 is overexpressed [12].

**Fig. 4:**
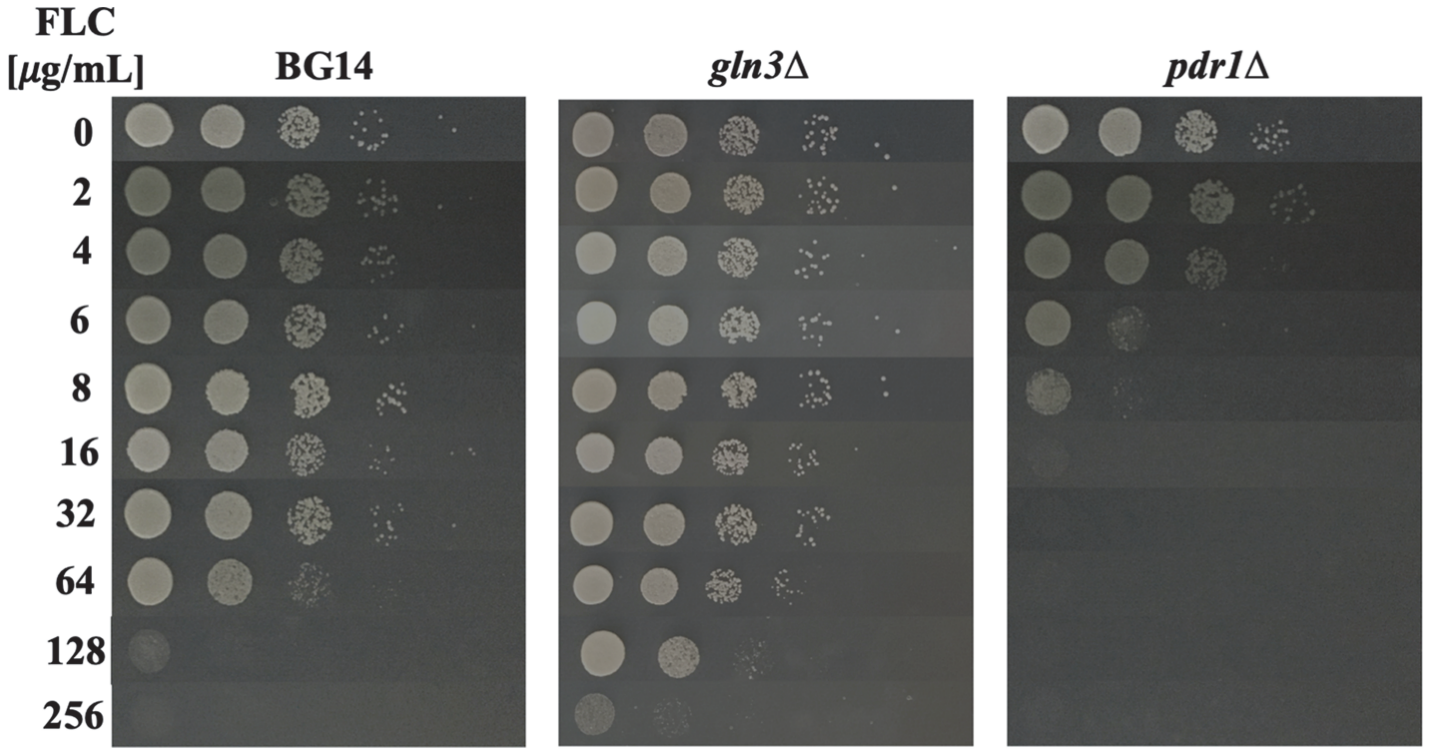
Gln3 negatively affects fluconazole resistance in *C. glabrata*. Ten-fold serial dilutions of BG14, *gln3*Δ and *pdr1*Δ strains were spotted in solid MM with ammonium as nitrogen source and buffered with MOPS (0.165M) to pH 6.5. Media were supplemented with a different concentration of fluconazole (0, 2, 4, 6, 8, 16, 128, 256 μg/ml^-1^). Plates were incubated for 48 h (BG14 and *pdr1*Δ strains) and 72 h (*gln3*Δ strain) at 30°C and photographed. A representative image of three independent experiments is depicted. FLC = fluconazole.

Pdr1 is the main transcriptional regulator involved in azole resistance and its absence correlates with high fluconazole susceptibility [18]. We considered that *PDR1* overexpression may decrease fluconazole susceptibility in a *gln3*Δ with respect to the BG14 strain. To test this, fluconazole susceptibility was evaluated in the BG14, *gln3*Δ and *pdr1*Δ strains. By using a spotted assay susceptibility, a breakpoint of 128 μg/ml^-1^ of fluconazole was determined for BG14 strain, while *gln3*Δ strain showed a decreased susceptibility of 256 μg/ml^-1^ (Fig. 4). As expected, *pdr1*Δ strain showed the highest susceptibility observed (Fig. 4), similar to what has been previously reported [18]. These results suggest a new role of Gln3 as a regulator of genes related to the detoxification of yeast cells after fluconazole exposure; however, more experiments are needed to determine if this transcriptional regulator is acting directly over these ABC transporters and its regulator.

## Discussion

The present study revealed that in *C. glabrata*, approximately 53.5% from the total detected genes (4,415 genes) are differentially expressed in BG14 and *gln3*Δ strains when cells grow in ammonium as a nitrogen source. Almost half of these DEGs (1,171 genes) were highly expressed in the BG14 compared to the *gln3*Δ strain. While 1,191 genes were overexpressed in the *gln3*Δ mutant compared to the BG14 strain. This high number of DEGs could be in part due to the main role of Gln3 as a NCR-regulator, as well as to a pleiotropic effect of Gln3, similar to what has been observed for *S. cerevisiae* [37, 38]. KEGG enrichment analysis of Up-DEG in BG14 strain compared to *gln3*Δ mutant mainly displayed gene expression associated with amino acid biosynthesis, replication and ribosome activity (Figs. 1C and 1E; Additional File 5). Meanwhile gene expression associations for Down-DEG in a *gln3*Δ compared to the parental strain were more diverse, including ABC transporters, starch and sucrose metabolism, and pyruvate metabolism (Figs. 1D and 1F; Additional File 5). It has been reported that when a *S. cerevisiae* parental strain grows in MM with ammonium as sole nitrogen source, there is an induction of genes related to amino acid transport, cell wall organization and biogenesis, glycolysis, lactate metabolism and glucose metabolism [37]. Although a transcriptome experiment of a parental vs a *gln3*Δ strain grown on ammonium has not been reported for *S. cerevisiae*, the enriched GO terms between the reported *S. cerevisiae* NCR genes (which are Gln3-dependent) [37] and the *C. glabrata* Gln3 positive-regulated genes (Up-DEG) herein identified are quite different. Only four enriched GO terms (response to chemical, carbohydrate metabolic process, generation of precursor metabolites and energy, and lipid metabolic process) are shared between *S. cerevisiae* NCR genes and *C. glabrata* Up-DEG. This suggests that *C. glabrata* Gln3 regulates a different set of genes and metabolic processes than those which have been already described to be regulated by *S. cerevisiae* Gln3.

Both, *S. cerevisiae* and *C. glabrata*, are able to use AAA as nitrogen sources, but only *C. glabrata* is able to grow when histidine is used as the sole nitrogen source [35]. Here, we found a positive role of Gln3 while growing on histidine and AAA (Fig. 2). Similarly, a positive role in the assimilation of different nitrogen sources has been observed for Gln3 orthologs in the closely related yeast *S. cerevisiae* and in the distant fungus C. albicans [39, 40]. In *S. cerevisiae*, the Ehrlich pathway provides nitrogen in the form of glutamate and 2-oxoglutarate through the transamination of AAA, mediated by the aromatic amino transaminases I and II (Aro8 and Aro9, respectively)[41]. *C. glabrata* also relies on the Ehrlich pathway for the assimilation of these amino acids; but in contrast to *S. cerevisiae*, only *ARO8* is highly inducible in the presence of AAA and histidine [35]. Our initial data suggested that the role of Gln3 in AAA and histidine assimilation could be through the transcriptional regulation of *ARO8* expression (Fig. 2 and Fig. S2). NCR target genes typically contain several 5’-GATA-3’ core sequences in their promoter region which can be recognized by the GATA family transcription factors, like Gln3 [14, 15]. In fact, three canonical GATA boxes (5’-GATAAG-3’) and seven 5’-GATWA-3’ boxes are located in the 1000-bp upstream region of *ARO8*. However, later experiments showed that Gln3 is dispensable for *ARO8* gene expression when cells were grown in AAA or histidine (Fig. S3). Four proteins have been implicated in the initial transamination step of the Ehrlich pathway, Aro8, Aro9, Bat1 and Bat2. Among these, only Aro8 and Aro9 are involved in the transamination of AAA and histidine in *C. glabrata* [35]. *ARO9* was not differentially expressed in our transcriptomic data and from the two remaining transaminases, only Bat1 coding gene was found to be repressed in Gln3 absence. However, it is unlikely that this aminotransferase is participating in the utilization of AAA and histidine. A possible explanation to the observed phenotype of the *gln3*Δ strain grown in AAA and histidine could be that this mutant has a general defect in amino acids transport. In this sense, we have previously shown that Gln3 regulates the expression of the general amino acid permease coding gene, *GAP1* [17], suggesting that the role of Gln3 in the assimilation of AAA or histidine could be independent of the Erlich pathway. This observation needs further investigation to determine whether Gln3 may directly regulate *ARO8* expression in MM with ammonium as nitrogen source.

The absence of Gln3 uncovered the overexpression of *CDR1*, *CDR2* and *PDR1*, which correlates with an increased fluconazole resistance (Fig. 4). These results are consistent with previous observations in which an increased expression of *PDR1*, *CDR1* and *CDR2* showed an increase in fluconazole resistance [9, 13, 42, 43]. From the two ABC transporters, Cdr1 and Cdr2, Cdr1 has been shown to play a discernible role in cellular detoxification from fluconazole [10]. It is a matter of importance to further investigate the role of Gln3 in *CDR1*, *CDR2* and *PDR1* regulation. One hypothesis could be that Gln3 is acting directly as a negative regulator of these genes; however, a role for Gln3 as a negative regulator has not been observed in *C. glabrata*, or even in its well-studied ortholog from *S. cerevisiae*. In our opinion, a more plausible hypothesis is that Gln3 is acting as an indirect negative regulator of *PDR1*, possibly by regulating a negative regulator; thus, Gln3 absence conducted to *PDR1* overexpression that in turn resulted in *CDR1* and *CDR2* overexpression, explaining *gln3*Δ resistance to fluconazole. Pdr1 overexpression has been previously observed to drive to an increased expression of *CDR1* and *CDR2*, while in accordance with our data, Pdr1 regular expression seems to only regulate *CDR1* (Fig. S4 and [12]). To our knowledge, this is the first report in which a main regulator of nitrogen assimilation has been associated with the drug efflux pumps in *C. glabrata*. These results pave the way toward clarifying the role of Gln3 as a possible indirect, negative regulator of *CDR1*, *CDR2* and *PDR1*. However, additional experiments are necessary to elucidate the specific role of Gln3 during fluconazole resistance for this fungal pathogen.

In conclusion, Gln3 is an important transcriptional factor in *C. glabrata* that regulates approximately 45% of the annotated transcripts in this yeast's genome. It also highlights the relevance in understanding the nitrogen assimilation pathway and antifungal resistance in *C. glabrata*.

## Supplemental Materials











Supplementary data for this paper are available on-line only at http://jmb.or.kr.

## Figures and Tables

**Table 1:** De novo transcriptome assembly and statistics of differentially expressed genes.

Number of libraries	6
Total raw reads	51,757,566
Total clean reads	44,837,461
Assembled transcripts	14,501
Coding transcripts	10,824
Non-coding transcripts	2,115
Annotated genes	4,415
Undetected genes	7
Total DEG	2,362
Up-DEG	1,171
Down-DEG	1,191
