# Supplementary material for: Transcriptome Analysis Unveils Gln3 Role in Amino Acids Assimilation and Fluconazole Resistance in Candida glabrata
Source: J Microbiol Biotechnol. 2021 Apr 21;31(5):659–66. doi: 10.4014/jmb.2012.12034 (PMC9705932; doi:10.4014/jmb.2012.12034)
Supplement: Supplementary file 1 [file jmb-31-5-659-supple1.pdf]

Supplementary information for

Transcriptome analysis unveils Gln3 role in amino acids assimilation and fluconazole resistance in *Candida glabrata*

Francisco J. Pérez-de los Santos (0000-0003-4753-3691)[1,2], Luis Fernando García-Ortega (000-0002-2021-7165)[1,3], Karina Robledo-Márquez (0000-0002-2737-5756)[2], Jesús Guzmán-Moreno (0000-0003-0237-6493)[2] and Lina Riego-Ruiz (0000-0002-2883-8871)[2*].

Affiliations

[1] Unidad de Genómica Avanzada, Laboratorio Nacional de Genómica para la Biodiversidad (Langebio), CINVESTAV.

[2] División de Biología Molecular, Instituto Potosino de Investigación Científica y Tecnológica A. C. (IPICYT).

[3] Departamento de Ingeniería Genética, Centro de Investigación de Estudios Avanzados del IPN (CINVESTAV).

[*] Corresponding author.

Table of contents

Figure S1	3
Figure S2	4
Figure S3	5
Figure S4	6
References	6
Additional File 1	7
Additional File 2	8
Additional File 3	8
Additional File 4	8
Additional File 5	8
KEGG_up_genes	
KEGG_down_genes	

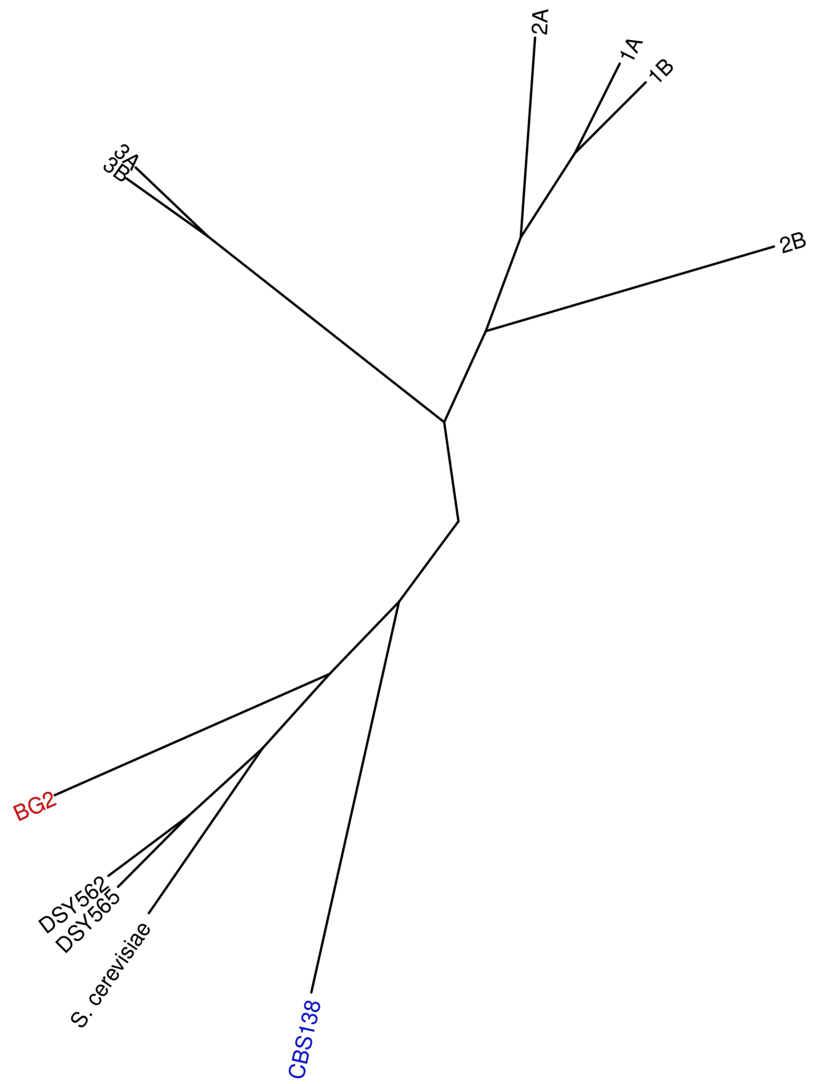

Figure S1. Phylogenetic tree based on 5,280 orthogroups obtained by STAG algorithm in OrthoFinder [1] of *Candida glabrata* strains sequenced and publicly available in the NCBI database. Saccharomyces cerevisiae S288c was used as outgroup. Tree was drawn with R and ggtree package [2].

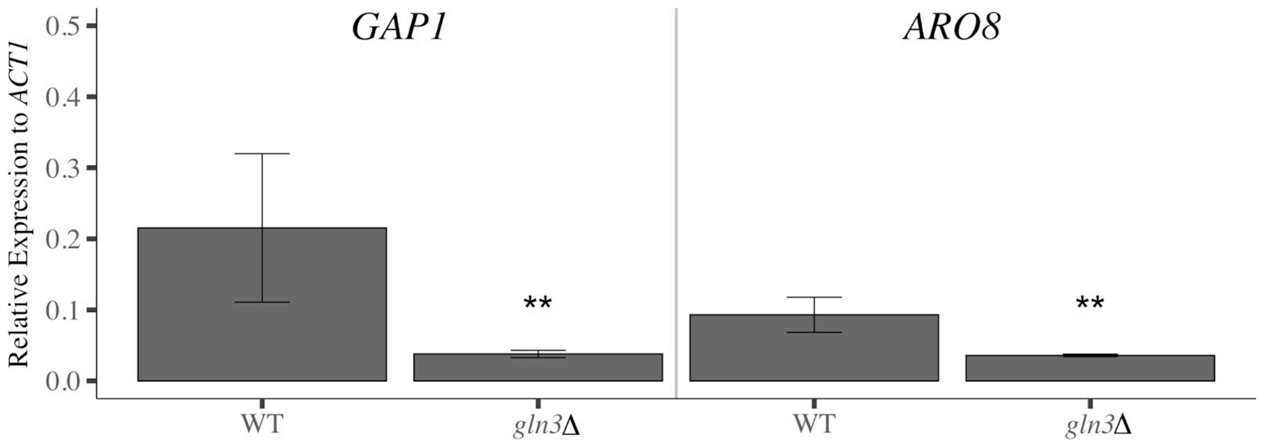

Figure S2. *ARO8* gene expression in MM with ammonium as sole nitrogen source. Gene expression of *ARO8* and *GAP1* was determined through qPCR of cDNA synthesized from 5 µg of total RNA extracted from yeast cultures on MM with ammonium as sole nitrogen source. *GAP1* gene expression was used as a control according to [17]. *ARO8* and *GAP1* expressions are relative to *ACT1* gene expression. Relative gene expression represents the mean of three independent experiments and two technical replicates ± standard deviation. ** Gene expression is significantly different from the one calculated in the WT strains, alpha < 0.01 in a two-tailed t-test.

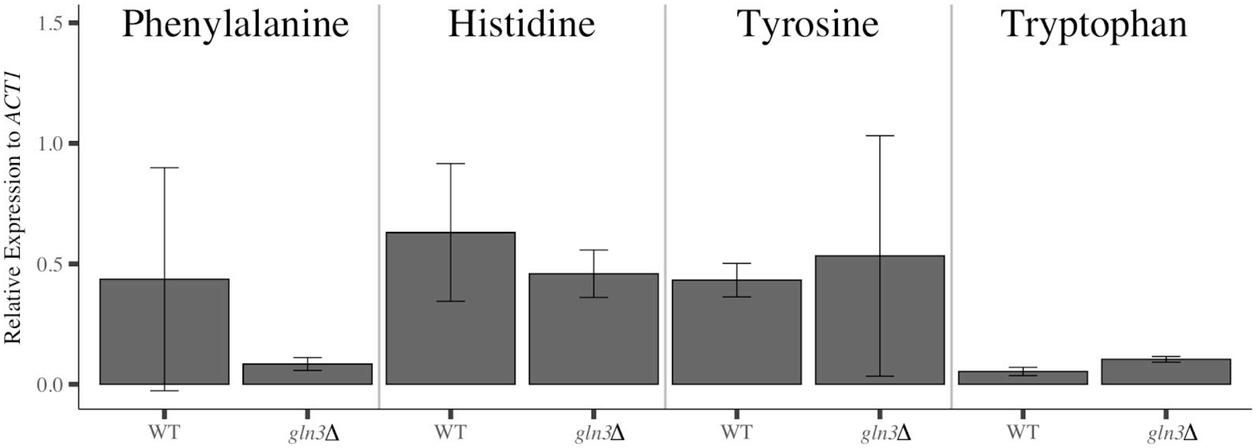

Figure S3. *ARO8* gene expression in MM with histidine or AAA as nitrogen sources. Gene expression of *ARO8* was determined through qPCR of cDNA synthesized from 5 μg of total RNA extracted from yeast cultures on MM with the indicated nitrogen sources. *ARO8* expression is relative to *ACT1* gene expression, and showed non-significant difference between the WT and *gln3*Δ strains in a two-tailed t-test. Relative gene expression represents the mean of three independent experiments and two technical replicates ± standard deviation.

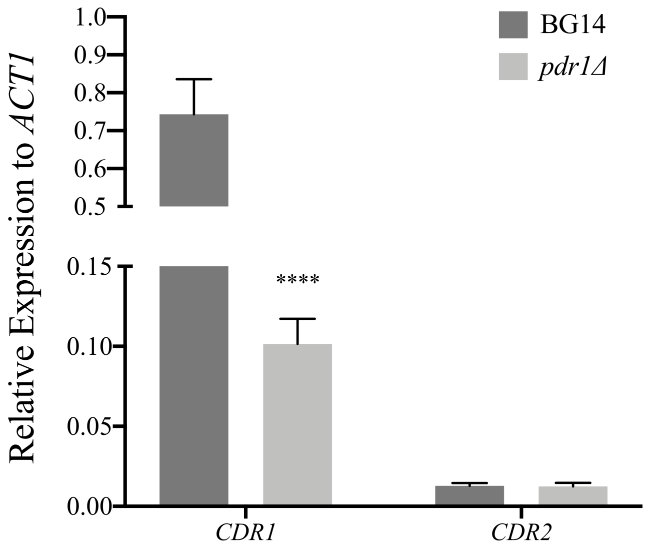

Figure S4. *CDR1* and *CDR2* gene expression in MM with ammonium as sole nitrogen source. Gene expression of *CDR1* and *CDR2* was determined in 5 µg of total RNA extracted from yeast cultures on MM with ammonium as sole nitrogen source. The reported gene expression is relative to *ACT1* and was measured through qPCR. Relative gene expression represents the mean of three independent experiments and six technical replicates ± standard deviation. **** Gene expression is significantly different from the calculated in the BG14 strain, alpha < 0.000001 in a two-tailed t-test.

References

1. Emms DM, Kelly S. 2019. OrthoFinder: phylogenetic orthology inference for comparative genomics. *Genome Biol*. 14: 238.

2. Yu G, Smith DK, Zhu H, Guan Y, Lam TT-Y. 2017. ggtree: an r package for visualization and annotation of phylogenetic trees with their covariates and other associated data. *Methods Ecol. Evol.* 8: 28–36.

Additional File 1. Primers used in the present study.

Systematic name	*S. cerevisiae* ortholog	Primer sequence (5' - 3')
CAGL0K12694g	*ACT1*	Fw CGCCGGTGACGATGCTCC<br>Rv CTTGGATTGAGCTTCGT
CAGL0M01760g	*CDR1*	Fw TCCACCAGATGCTAACCCAG<br>Rv CTCGTTGGACATCCACTCCA
CAGL0F02717g	*CDR2/PDR15*	Fw ATGCTGGGATAACGCTACGA<br>Rv GAGATTGTAGGCGTCTTGCG
CAGL0A00451g	*PDR1*	Fw ACTGCATCTCCCTTATCGGG<br>Rv ATGGAACGTTGATCTCCCCA
CAGL0L03267g	*GAP1*	Fw CACTACCTATGCCACGAGATTC<br>Rv TCACCGTAACCTTTCACACC
CAGL0G01254g	*ARO8*	Fw TCTTTCTTGGTGGTGGTCTTC<br>Rv GTCTTCTTGAGAGCCGGTTATAG

Additional File 2. Genes with expression evidence.

Additional File 3. Gene expression levels.

Additional File 4. Differentially expressed genes.

Additional File 5. Genes in KEGG pathways.

KEGG_up_genes

KEGG_down_genes
